# Supplementary material for: Cost-effectiveness of one-year adjuvant trastuzumab therapy in treatment for early-stage breast cancer patients with HER2+ in Vietnam
Source: PLoS One. 2024 Mar 15;19(3):e0300474. doi: 10.1371/journal.pone.0300474 (PMC10942069; doi:10.1371/journal.pone.0300474)
Supplement: S1 Text — (DOCX) [file pone.0300474.s005.docx]

# ELECTRONIC SUPPLEMENT

# Cost-effectiveness analysis of Trastuzumab in treatment for early-stage breast cancer patients with HER2+ in Vietnam

**Text S1: Cost analysis**

This section outlines the steps used to conduct the cost analysis of adjuvant trastuzumab therapy, cost for treating cardiac event and cost for other health states.

**S1.1. Cost analysis of adjuvant trastuzumab therapy**

Table S1.1 presents a summary of the different cost items associated with one-year adjuvant trastuzumab therapy. The total cost of each cost item is (broadly speaking) the product of unit cost, quantity and (when applicable) probability. Resource use parameters and unit costs that were used to enumerate these cost items are presented in Table S2 below. A detailed summary of the method used to cost the one-year adjuvant trastuzumab therapy is provided below.

***Table S1.1. Cost of one year adjuvant trastuzumab therapy***

| Cost items | Unit cost  (VND) | Quantity  Per 1 patient | Probability | Expected cost |
| --- | --- | --- | --- | --- |
| **A. Cost of diagnosis test for HER2** |  |  |  |  |
| A1. IHC test | 172,169 | 1 | 1.00 | 172,169 |
| A2. FISH test | 3,212,169 | 1 | 0.16 | 513,947 |
| A3. Total (A3=A1+A2) |  |  |  | 686,116 |
| **B. Cost of trastuzumab (drug only)** |  |  |  |  |
| B1. Herceptin 150mg (vial) | 15,550,710 | 51 | 0.68 | 541,047,891 |
| B2. Herceptin 440mg (vial) | 45,596,775 | 17 | 0.32 | 246,336,759 |
| B3. Total (B3=B1+B2) |  |  |  | 787,384,650 |
| **C. Cost for administration of trastuzumab** |  |  |  |  |
| C1. General check-up (visits) | 38,115 | 17 | 1.00 | 647,955 |
| C2. Para-clinical tests (times) | 371,834 | 17 | 1.00 | 6,321,178 |
| C3. Cardiac risk assessment test (times) | 151,294 | 4 | 1.00 | 605,176 |
| C4. Other materials, diffusion, and supportive drugs (times) | 29,898 | 17 | 1.00 | 508,266 |
| C5. Hospitalization (days) | 150,157 | 34 | 1.00 | 5,105,338 |
| C6. Other direct medical costs (days) | 407,442 | 34 | 1.00 | 13,854,028 |
| C7. Total direct medical cost  (C7=C1+C2+C3+C4+C5+C6) |  |  |  | 27,040,970 |
| C8. Total direct non-medical cost | 168,358 | 34 | 1.00 | 5,724,180 |

**S1.1.A. Cost of diagnosis test for HER2**

Based on expert opinion and the most updated national treatment guideline in Vietnam (i.e., Decision 3128/QD-BYT approved the “Guideline on Breast cancer diagnosis and treatment” in 2020), it was assumed that 100% of patients have an immunohistochemical (IHC) for detecting HER2 status. Among patients taking the IHC test, only 16% of the patients (i.e., 5% of the patients with IHC3+ results, 4% with IHC2+ results, and 7% with IHC1+/- results) were assumed to proceed with the FISH test.

The unit cost of IHC was estimated to include the cost from the government (due to subsidy), i.e., the total of input parameters 1.5, 1.6, and 1.7 in Table S2 and the cost from payers (reimbursement from social health insurance (SHI) and copayment from patients), i.e., input parameter 1.3 in Table S2. The unit cost of the FISH test was similarly calculated.

**S1.1.B. Cost of trastuzumab (drug only)**

Based on expert opinion and the most updated national treatment guideline in Vietnam (i.e., Decision 3128/QD-BYT approved the “Guideline on Breast cancer diagnosis and treatment” in 2020), we calculated trastuzumab cost for a 3-weekly dose with one starting does of 8 mg/kg and 17 sequent does of 6 mg/kg for each patient. The weight of patients (i.e., input parameter 1.1 in Table S2) was assumed to be equal the average weight of patients from patient survey in 2015 (n=56) and quality of life survey in 2014 (n=309). There are two types of Herceptin vial, which are 150mg vial and 440mg vial. Based on the mg of Herceptin needed per each administration (i.e., dose mutiplied with average patient weight), we estimated the quantity of vials needed to purchase for each patients per year. Unit cost of Herceptin 150mg/440mg vial of were collected from Vietnam Drug Administration (DAV) website. Doctors and patients can choose to use the Herceptin 150mg vial or 440mg vial based on the availibility of the vials in the market. Thus, based on the total number of 150mg and 440mg vials available in the market (collected from DVA website), we estimated the proportion of each vial to estimate the one-year trastuzumab cost.

**S1.1.C. Cost for administration of trastuzumab**

Total cost for administration of trastuzumab can be devided in to direct medical cost (C7) and direct non-medical cost (C8) as depicted in Table S1.1.

Direct medical cost (C7) included six cost items: Cost of general check-up (C1); Cost of para-clinical tests, including CBC, SGOT, SGPT, Alkaline phosphatase, Bilirubin, Albumin, Ure, Acid Uric, Creatinin (C2), cost of cardiac risk assessment test, including echocardiogram and electrocardiogram (C3); Cost of other materials, diffusion and supportive drugs required per each administration of trastuzumab (C4); cost of hospitalization (C5); and other direct medical costs, such as other additional drugs, medical services, nursing services, etc., (C6).

Resource use parameters for each cost item were collected based on expert opinion and the most updated national treatment guideline in Vietnam (i.e., Decision 3128/QD-BYT approved the “Guideline on Breast cancer diagnosis and treatment” in 2020). The values for resource use parameters are presented in Table S2. For example, based on Decision 3128, for a 3-weekly dose administration approach, patients needed to administer trastuzumab 17 times (one starting dose and 16 sequent doses). Each time of administration, patients need to stay hospitalized for 2 days and re-take a set of basic para-clinical tests. It is also needed to perform cardiac risk assessment every 3 months, i.e., 4 times per year (Parameter 1.43 in Table S2).

Similar to the methods of calculating the unit cost of IHC/FIST test, the unit cost of other health services, including general check-ups (C1); para-clinical tests (C2), echocardiogram and electrocardiograms for cardiac risk assessment (C3), hospitalization (C5) was the sum of cost from the government and cost from the payers.

Costs from the government (i.e., government subsidy for labor cost, depreciation cost, and overhead cost) were collected from a costing study in the four large oncology hospitals in 2014 [16] in Vietnam (including Vietnam National Cancer Hospital, Hanoi Oncology Hospital, Da Nang Oncology Hospital, Ho Chi Minh City Oncology Hospital). The study followed the standard activity-based costing approach to calculate the full cost of each service performed. See Table S2 for detailed unit cost parameters.

Fees for laboratory tests, general examination, and inpatient beds were collected from the current validated national health service fee schedule (Circular 13/2019/TT-BYT approved by the Vietnam Ministry of Health since 20/09/2019). Fees for services presented the cost from payers, i.e., SHI and patients. See Table S2 for detailed unit cost parameters.

Unit cost of other materials, diffusion and supportive drugs required per each administration of trastuzumab (C4); and other direct medical costs, such as other additional drugs, medical services, nursing services, etc., (C6); and direct non-medical costs were collected based on patients surveys.

**S1.2. Cost analysis of congestive heart failure:**

Table S1.2 presents a summary of the different cost items associated with congestive heart failure. The total cost of each cost item is (broadly speaking) the product of unit cost, quantity and (when applicable) probability. Resource use parameters and unit costs that were used to enumerate these cost items are presented in Table S2 below. A detailed summary of the method used to cost the congestive heart failure is provided below.

***Table S1.2. Cost of congestive heart failure:***

| Cost items | Unit cost  (VND) | Quantity  Per 1 patient | Probability | Expected cost |
| --- | --- | --- | --- | --- |
| **D. Cost of inpatient treatment** |  |  |  |  |
| D1.Para-clinical tests (times) | 371,835 | 2 | 1.00 | 743,669 |
| D2.X-ray (times) | 113,573 | 2 | 1.00 | 227,145 |
| D3.Electrocardiogram (times) | 60,722 | 2 | 1.00 | 121,443 |
| D4.Hospitalization (days) | 150,157 | 14 | 1.00 | 2,102,199 |
| D5.Drug cost |  |  |  |  |
| Furocemid (mg) | 79.32 | 700 | 1.00 | 55,527 |
| Spironolacton (Verospiron) (mg) | 74.91 | 356.75 | 0.50 | 13,362 |
| Spironolacton (Diulacton) (mg) | 51.20 | 262.50 | 0.50 | 6,720 |
| Lisinopril (mg) | 375 | 426.25 | 0.33 | 53,281 |
| Enalapril (mg) | 145 | 220.80 | 0.33 | 10,672 |
| Ramipril (mg) | 724 | 83.13 | 0.33 | 20,062 |
| Telmisartan (mg) | 111 | 840 | 0.50 | 46,620 |
| Valssartan (mg) | 76 | 3362 | 0.50 | 127,756 |
| D6.Direct non-medical cost | 168,358 | 14 | 1.00 | 2,357,015 |
| **E. Cost of outpatient treatment** |  |  |  |  |
| Furocemid (mg) | 79.32 | 4500 | 1.00 | 356,959 |
| Spironolacton (Verospiron) (mg) | 74.91 | 1687 | 0.50 | 63,186 |
| Spironolacton (Diulacton) (mg) | 51.20 | 1687 | 0.50 | 43,187 |
| Lisinopril (mg) | 375 | 2925 | 0.33 | 365,625 |
| Enalapril (mg) | 145 | 1494 | 0.33 | 72,210 |
| Ramipril (mg) | 724 | 506 | 0.33 | 122,114 |
| Telmisartan (mg) | 111 | 5400 | 0.50 | 299,700 |
| Valssartan (mg) | 76 | 22860 | 0.50 | 868,680 |

Assuming that after the event of CHF, the patient was treated with the mean hospitalization time of two weeks (14 days) and the outpatients’ management duration of three months (90 days). We identified the cost items based on the American Heart Association treatment guideline (also followed by Vietnam physicians as current practice).

Cost of inpatient treatment included: Para-clinical tests (D1); X-ray (D2); Electrocardiogram (D3); Hospitalization (D4); Drug cost (D5) and direct non-medical cost (D6). The cost quantities were estimated based on consultation with a physician expert. Unit costs for D1, D2, D3, D4, and D6 were estimated similarly to cost elements A and C as explained above. Drug costs (D5) were collected from the Vietnam Drug Administration (DAV) website. The dose for each drug was based on the American Heart Association treatment guideline (also followed by Vietnam physicians as current practice). It was assumed that the drugs in the same group (i.e., Verospiron and Diulacton; lisinopril, enalapril, and ramipril; telmisartan and valsartan) have an equal chance to be used. Other drugs, such as beta-blockers, digoxin, vasodilator group, antibiotics, and anticoagulants as needed depending on the accompanying pathology were not included.
